# The effect of Hypnotherapy-based interventions on improving women’s experience of pregnancy, childbirth and postpartum: A narrative review

**DOI:** 10.1192/j.eurpsy.2023.2017

**Published:** 2023-07-19

**Authors:** F. Seitz, M. Pourasghar, J. Ganji

**Affiliations:** 1 M.Sc. Student, Counseling in Midwifery, Student Research Committee, Mazandaran University of Medical Sciences; 2 Department of Psychiatry, Psychiatric and Behavioral Sciences Research Center, Addiction Institute, Mazandaran University of Medical Sciences; 3 Department of Reproductive and Midwifery Health, Sexual and Reproductive Health Research Center, Mazandaran University of Medical Sciences, Sari, Iran, Islamic Republic Of

## Abstract

**Introduction:**

Hypnotherapy has been increasingly used in recent years in healthcare, with several applications during pregnancy, labor, birth and the postpartum.

**Objectives:**

This review was performed to assess the effects of Hypnotherapy before, during and after pregnancy.

**Methods:**

A narrative review methodology using keywords determined by the Medical Subject Headings (MeSH) thesaurus was adopted in this study. For this purpose, the databases of PubMed, Scopus,Web of Science, GoogleScholar, and Scientific Information Database (SID) were searched using the keywords of “Hypnosis, Hypnotherapy, Pregnancy, Labor, and Childbirth ” from March11 to April 5, 2022; and finally, the related articles published from 2000 to 2022 were retrieved.

**Results:**

According to the findings, the effects ofHypnotherapy on pregnancy and delivery and postpartumoutcomes were classified into several categories as the following: Hypnotherapy-based interventions improve childbirth experience, with less anxiety, increased satisfaction, fewer birth interventions, more postnatal well-being and better childbirth experience overall. Hypnotherapy may reduce the overall use of analgesia during labour, but not epidural use.Hypnotherapy intervention during pregnancy aided in reducing physical and psychological symptoms during pregnancy.

**Conclusions:**

With reference to the related literature on this subject matter, women can safely pursue hypnotherapy during pregnancy, labor, birth and the postpartum. Hypnotherapy can be presented as a technique enabling patients to have a positive birth experience; however, high quality trials are needed to demonstrate its complete efficacy.

**Disclosure of Interest:**

None Declared

